# A Metabolic Reprogramming of Glycolysis and Glutamine Metabolism Is a Requisite for Renal Fibrogenesis—Why and How?

**DOI:** 10.3389/fphys.2021.645857

**Published:** 2021-03-17

**Authors:** Timothy D. Hewitson, Edward R. Smith

**Affiliations:** ^1^Department of Nephrology, The Royal Melbourne Hospital (RMH), Melbourne, VIC, Australia; ^2^Department of Medicine-RMH, The University of Melbourne, Melbourne, VIC, Australia

**Keywords:** fibroblast, fibrosis, glycolysis, glutaminolysis, metabolic, metabolism, priming, TGF-β1

## Abstract

Chronic Kidney Disease (CKD) is characterized by organ remodeling and fibrosis due to failed wound repair after on-going or severe injury. Key to this process is the continued activation and presence of matrix-producing renal fibroblasts. In cancer, metabolic alterations help cells to acquire and maintain a malignant phenotype. More recent evidence suggests that something similar occurs in the fibroblast during activation. To support these functions, pro-fibrotic signals released in response to injury induce metabolic reprograming to meet the high bioenergetic and biosynthetic demands of the (myo)fibroblastic phenotype. Fibrogenic signals such as TGF-β1 trigger a rewiring of cellular metabolism with a shift toward glycolysis, uncoupling from mitochondrial oxidative phosphorylation, and enhanced glutamine metabolism. These adaptations may also have more widespread implications with redirection of acetyl-CoA directly linking changes in cellular metabolism and regulatory protein acetylation. Evidence also suggests that injury primes cells to these metabolic responses. In this review we discuss the key metabolic events that have led to a reappraisal of the regulation of fibroblast differentiation and function in CKD.

## Introduction

While the kidney can recover from acute injury, persistent and/or severe injury results in the chronic accumulation of scar tissue (fibrosis) and progressive renal failure. Understanding the mechanisms that regulate the transition from acute kidney injury to chronic kidney disease (CKD) is important, because once fibrosis is initiated it can be extremely difficult to switch off or reverse (Hewitson, 2009; Hewitson et al., 2017a). A defining characteristic of this transition is a maladaptive repair and a persistent activation of fibroblast-like cells (Darby and Hewitson, 2007). These cells are the major source of the excess extracellular matrix (ECM) proteins in a process that is inherently similar in different organ pathologies.

## What is a Fibroblast?

Single cell sequencing has highlighted the considerable heterogeneity of the fibroblast population in the kidney (Wu et al., 2019). One of the most vexed questions in fibrosis research is therefore—how do we define a fibroblast? Potentially fibroblasts include any ECM-producing cell in the connective tissue stroma. Consistent with their diversity, multiple cellular origins for the fibroblast have been suggested including epithelial- and endothelial-to-mesenchymal transition, infiltration of bone marrow precursors and macrophages (Duffield et al., 2013), along with a resident quiescent mesenchymal stem cell precursor, often simply referred to as a pericyte (Chou et al., 2020).

## Role of the Myofibroblast

It has repeatedly been shown that a population of activated fibroblasts can be identified by *de novo* expression of α-smooth muscle actin (αSMA), a protein normally associated with smooth muscle cells (Darby and Hewitson, 2007). These cells display properties of both fibroblasts and smooth muscle cells including prodigious synthesis of ECM and contractile functions through the formation of actin-myosin fibers (Tomasek et al., 2002). Interstitial myofibroblasts are a feature of both primary and secondary tubulointerstitial pathologies in the kidney.

The myofibroblast was first described in skin wound healing where its transient presence in granulation tissue is responsible for repair and contraction of the wound area to facilitate healing. Based on this, it is often assumed that they have a similar acute repair role in the kidney in response to injury. Accordingly, some have postulated that these cells are initially recruited to synthesize ECM in order to stabilize injured tubules (Kaissling et al., 2013). Regardless, their ongoing presence in the kidney, as elsewhere, results in scar tissue formation and contracture.

## Activation of Fibroblasts

The initial differentiation of fibroblasts to so-called myofibroblasts is a process driven by cytokines/growth factors released by injured parenchyma and infiltrating inflammatory cells (Darby and Hewitson, 2007). While a number of pro-fibrotic influences exist, transforming growth factor-β1 (TGF-β1) has consistently been shown to be the pre-eminent fibrogenic signal (Meng et al., 2016).

### Fibroblasts Can Act Autonomously

During fibrosis, fibroblasts begin to act autonomously and independently of tubular and leukocyte inflammation (Leaf and Duffield, 2017). Indeed, recently Buhl et al. have elegantly shown that activation of fibroblasts *per se* is sufficient to drive progressive fibrosis and systemic features of CKD (Buhl et al., 2020), independent of any surrounding tubular and interstitial pathologies.

This process seems particularly pertinent to TGF-β1, which is secreted as a latent protein bound to adjacent ECM and with activation on release from the latency complex. Thus, TGF-β1 synthesized by tubules can't simply diffuse across basement membranes to activate interstitial fibroblasts (Venkatachalam and Weinberg, 2015). This key difference between TGF-β1 and many other growth factors and cytokines, highlights an important autocrine regulatory mechanism. Myofibroblasts isolated from hypertrophic scars exhibit a stably differentiated and contractile phenotype *via* a TGF-β1-dependent autocrine loop involving focal adhesion proteins (Dabiri et al., 2008). Here TGF-β1 is activated by myofibroblasts “pulling” on the TGF-β1 complex to release the active form. This dependence on generating tissue tension demonstrates that feed-forward loops can drive myofibroblast differentiation (Dabiri et al., 2008). TGF-β1 can also operate as a feed forward loop to amplify the effects of other novel pro-fibrotic growth factors such as FGF23 (Smith et al., 2017a,b).

## Metabolic Regulation of Fibroblast Function

While a major focus of fibrosis research has been on the transcriptional regulation of collagen synthesis, we now also appreciate that TGF-β1 is a trigger for a metabolic reprogramming that is needed for fibroblast synthesis and contraction.

Physiological cellular metabolism consists of specific metabolic reactions involving conversion of a carbon source into the building blocks needed for macromolecule biosynthesis, energetics and cellular homeostasis (see Altman et al., 2016; O'Neill et al., 2016). Alterations in cellular metabolism, including the so-called “Warburg effect,” are thought to play an essential role in the acquisition and maintenance of a malignant phenotype in tumor cells (Chen et al., 2018). Fibroblasts in a number of organs undergo similar dramatic metabolic changes during activation that are necessary to meet the increased bioenergetic and biosynthetic demands of mitogenesis and ECM synthesis (fibrogenesis) [reviewed in (Xie et al., 2015; Para et al., 2019; Zhao et al., 2020)] (Table 1). Because TGF-β1 has been identified as the specific orchestrator in many cases (Negmadjanov et al., 2015; Ding et al., 2017; Si et al., 2019; Barcena-Varela et al., 2020; Bates et al., 2020; Henderson et al., 2020; Smith and Hewitson, 2020), the molecular events underlying these global adaptations are of particular mechanistic interest.

**Table 1:** Representative *in vitro* examples of metabolic adaptations in fibroblast-like cells.

**Organ**	**Cell**	**Metabolic adaptations**	**References**
Skin	Normal human skin and keloid fibroblasts	↑glycolysis in keloid fibroblasts vs. normal fibroblasts	Li et al., 2018
Liver	Human hepatic stellate cell line	TGF-β1 ↑glycolysis ↓OxPhos	Bates et al., 2020
Liver	Hepatic stellate cell line (LS2)	TGF-β1 ↑glycolysis ↓OxPhos	Barcena-Varela et al., 2020
Lung	Normal human and IPF lung Fibroblasts	↑glycolysis in IPF	Xie et al., 2015
Peritoneum	Human mesothelial cell line (MCT-5A)	TGF-β1 ↑glycolysis ↓OxPhos	Si et al., 2019
Kidney	Rat interstitial fibroblast	TGF-β1 ↑glycolysis ↓OxPhos↑Glutaminolysis	Smith and Hewitson, 2020
Lung	Normal human and IPF lung fibroblasts	↑Gls mRNA in IPF vs. normal	Choudhury et al., 2020
Lung	Human Lung fibroblasts	Absence of glutamine ↓TGF-β1 stimulated collagen and αSMA	Hamanaka et al., 2019
Lung	Human Lung fibroblasts	TGF-β1 ↑Gls and glutamine consumption	Ge et al., 2018

### Rewiring Glycolysis

**Figure 1:**
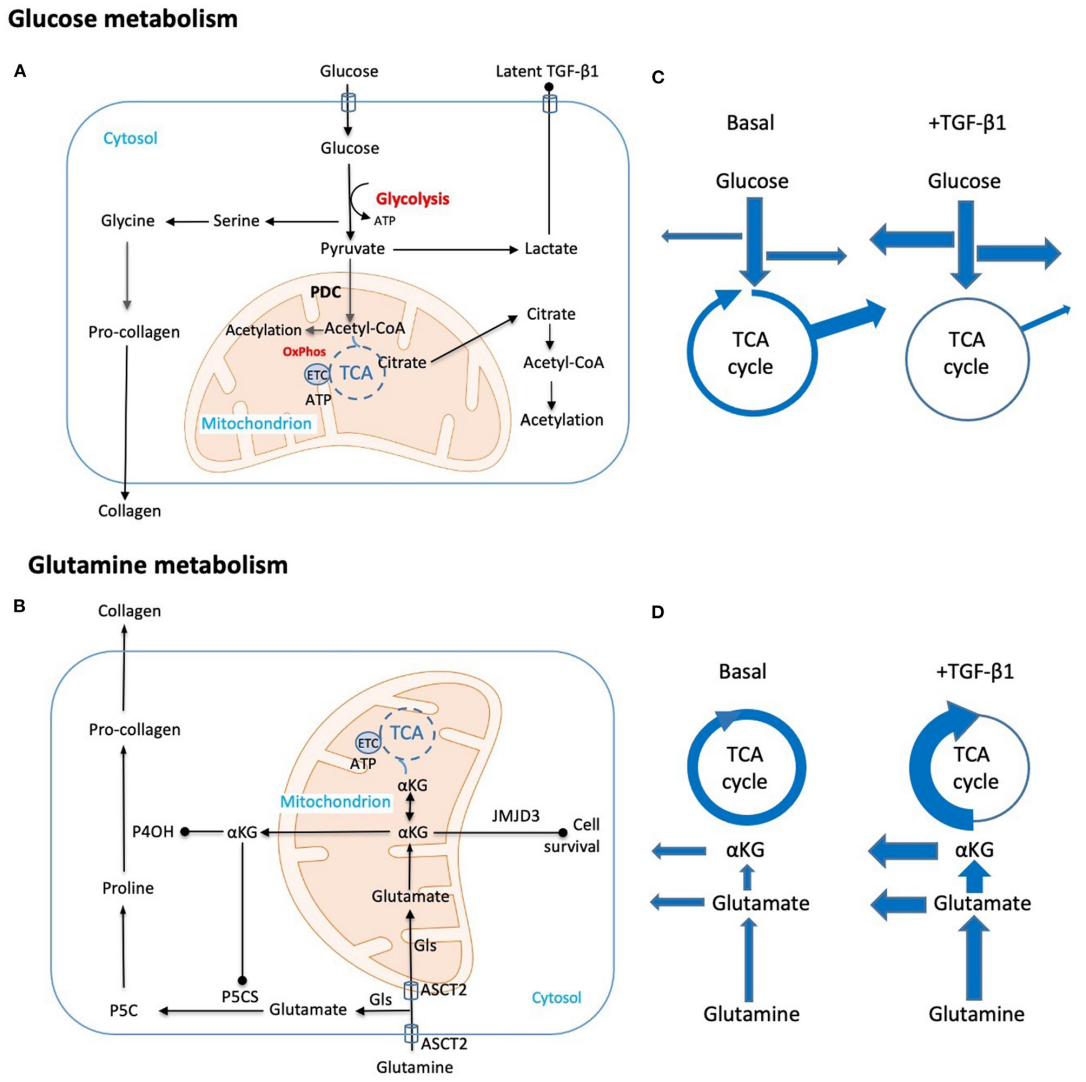
Schematic representation of key metabolic adaptations in fibroblasts to support collagen synthesis. Metabolic and biosynthetic fates of **(A)** glucose and **(B)** glutamine highlight potential synthetic (arrow head) and regulatory (solid dot) functions in fibrogenesis. **(C,D)** Putative changes in metabolic flux caused by TGF-β1 are diagrammatically indicated by changes in arrow thickness. These include **(C)** a shift from oxidative phosphorylation to glycolysis with commensurate increases in amino acid and nucleotide synthesis and a reduction in Acetyl-CoA generation. Export of the end product lactate may also be pro-fibrotic as local changes to pH contribute to activation of latent TGF-β. **(D)** Parallel hypothesized TGF-β1 induced changes in glutamine metabolism both supplement amino acid synthesis and TCA intermediates lost through metabolic shifts in glucose metabolism. Key: Acetyl-CoA, Acetyl coenzyme-A; ASCT2, alanine-serine-cysteine transporter 2; αKG, α-ketoglutarate; ATP, adenosine triphosphate; ETC, electron transport chain; Gls, glutaminase; JMJD3, Jumonji domain-containing protein D3; OxPhos, oxidative phosphorylation; P4OH, prolyl-4-hydroxylase; P5CS, pyrroline-5-carboxylate synthase; P5C, pyrroline-5-carboxylate; PDC, pyruvate dehydrogenase complex; TCA, tricarboxylic acid cycle; TGF-β1, transforming growth factor-β1.

Principal amongst the metabolic changes induced by TGF-β1 is an increase in glycolytic flux, despite the availability of oxygen (aerobic glycolysis), and uncoupling from oxidative phosphorylation in the mitochondria (Figure 1A) (Ding et al., 2017; Smith et al., 2019; Smith and Hewitson, 2020). This metabolic shift is also supported clinically by a metabolomic sub-analysis of the Chronic Renal Insufficiency Cohort (CRIC) study showing that a decrease in the tricarboxylic acid (TCA) cycle intermediates, citrate and aconitate, correlate with declining eGFR in diabetic kidney disease (DKD) (Kwan et al., 2020).

There are several reasons why this redirection of carbon may be functionally significant in fibrosis. Although glycolysis is less efficient at producing adenosine triphosphate (ATP) than oxidative phosphorylation coupled with the electron transport chain, glycolysis produces ATP faster (Para et al., 2019). Additionally, this means that carbon can be redirected to other biosynthetic pathways (e.g., pentose phosphate pathway and nucleotide synthesis etc.), and critically with respect to collagen production, enhances generation of non-essential amino acids, such as glycine, which constitutes 35% of all amino acids in collagen (Nigdelioglu et al., 2016). The ultimate conversion of pyruvate to lactate, and its export, results in acid-induced TGF-β1 activation *in vitro* (Kottmann et al., 2012, 2015). Indeed, genetic or pharmacologic approaches that block glycolysis decrease contraction and reduce TGF-β1-induced αSMA and collagen expression in the fibroblastioc IMR-90 cell line (Bernard et al., 2015). In the mouse, blockade of glycolytic flux with shikonin, an inhibitor of pyruvate kinase M2, ameliorates fibrosis after unilateral ureteric obstruction (Wei et al., 2019).

### Metabolic Switches

A more in depth analysis of the metabolic reprogramming in renal fibroblasts suggests that inactivation of the pyruvate dehydrogenase complex (PDC) is an important metabolic switch in maintaining a chronically activated fibrogenic state (Smith and Hewitson, 2020). The PDC catalyzes a series of rate-limiting reactions involved in the oxidative decarboxylation of pyruvate to acetyl-CoA (Figure 1). Analysis of the canonical pathways differentially regulated by TGF-β1 identified inhibition of acetyl-CoA biosynthesis *via* inactivation of PDC as a potential metabolic regulator of fibroblast activation in cells derived from fibrotic kidneys (Smith and Hewitson, 2020). Accordingly, TGF-β1 induces a profound reduction in cellular acetyl-CoA stores. Inhibition of PDC through increased activity of pyruvate dehydrogenase kinases (PDK) also leads to a fibrogenic Warburg-like phenotype in cardiac fibroblasts (Tian et al., 2020). Other direct targets of TGF-β1 include the glycolytic enzymes phosphofructokinase (PFK) (Calvier et al., 2017) and hexokinase (Yin et al., 2019) which catalyze key regulatory steps in the conversion of glucose to pyruvate.

### Metabolic Regulation of Protein Acetylation

Augmented glycolysis also has more widespread implications (Ghosh-Choudhary et al., 2020). Acetyl-CoA is not only a substrate for the TCA cycle and other biosynthetic pathways (e.g., fatty acid synthesis), but it is also the obligatory acetyl donor for regulatory protein lysine acetylation (Sivanand et al., 2018), thus providing a potential direct link between changes in cell metabolism and protein function (Kori et al., 2017; Weinert et al., 2018).

Acetylation of amino acid residues has regulatory functions at various steps between transcription and protein degradation (Spange et al., 2009). These include changes in both the size and electrostatic charge of amino acid side chains, altered enzyme activity through changing binding preference, competitive interaction with other modifications and finally, the creation of new protein docking sites (Spange et al., 2009).

Under physiological conditions, glucose accounts for up to 90% of the acetyl-CoA pool (Kamphorst et al., 2014), with the majority being generated in the mitochondria through the decarboxylation of pyruvate *via* PDC. Mitochondrial acetyl-CoA is exported as citrate, and converted back into acetyl-CoA, and used for acetylation within both the cytoplasm and nucleus. Nuclear acetyl-CoA may also be supplemented *in situ* by nuclear PDC (Sutendra et al., 2014).

To date, most interest in acetylation has focused on the role of nuclear histone acetylation in epigenetic regulation of transcription. However, the significance of protein acetylation extends well beyond histones (Choudhary et al., 2009, 2014) with proteomic analysis revealing that in excess of 2,000 proteins can be acetylated in the kidney (Lundby et al., 2012). Consistent with a reduction in global acetyl-CoA levels, it has been shown that TGF-β1 produces a corresponding reduction in lysine acetylation of many, as yet unidentified, proteins in rat kidney fibroblasts (Smith and Hewitson, 2020), thus extending our earlier finding that TGF-β1 regulates histone H3 acetylation (Hewitson et al., 2017b; Smith et al., 2019). These effects were ameliorated when PDC was maintained in an activated state with dichloroacetate, a reversible inhibitor of inactivating PDKs (Smith and Hewitson, 2020).

Kinetic studies of acetylation show that it is rapidly reversible (Kori et al., 2017; Weinert et al., 2018) suggesting that it can act as a modification-based switch, analogous to that seen with phosphorylation. A noteworthy example of this exists in the regulation of fibrosis. Canonical TGF-β1 signaling is *via* the transcription factors Smad 2, 3, and 4, in turn counter balanced by actions of the inhibitory Smad 7 (Meng et al., 2016). Acetylation protects Smad 7 from degradation by ubiquitination. Conversely, deacetylation increases ubiquitination and degradation, thus removing a well-described anti-fibrotic brake. In this context, findings that TGF-β1 is responsible for the deacetylation of Smad 7 (Fukasawa et al., 2004) establish a functional significance.

### Glutamine Metabolism

Enhanced glycolytic flux alone cannot meet the high metabolic demands of fibroblasts and increased carbon supply through alternate pathways is needed to support biosynthetic requirements. The maintenance of high levels of glutamine in the blood through diet and synthesis by muscle and other organs provides a ready source of carbon and nitrogen to support cell growth. Physiologically glutamine in the kidney is metabolized most in tubules. As in tumor cells, along with an increase in glycolysis, glutamine metabolism is also increased *in vitro* by TGF-β1 in lung (Bernard et al., 2018; Ge et al., 2018; Hamanaka et al., 2019) and dermal (Henderson et al., 2020) fibroblast-like cells. Clinically, epidemiological analyses have shown that a decrease in urine glutamine is predictive of progression from moderate to severe albuminuria in DKD (Pena et al., 2014), consistent with increased use in biosynthetic pathways.

Glutamine is transported into the cell by the solute carrier Slc1A5 (ASCT2), where it is converted into glutamate by glutaminase (Gls 1 or Gls 2 depending on the tissue). Augmented glutamine consumption in lung myofibroblasts appears largely driven by elevated levels of Gls1 (Bernard et al., 2018; Ge et al., 2018). Proline, which together with glycine accounts for 57% of amino acid residues in collagen (Li and Wu, 2018), is generated directly from glutamate *via* pyrroline-5-carboxylate (P5C) (Li and Wu, 2018) in the cytosol, while in the mitochondrion glutamine can be converted to α-ketoglutarate (αKG), an intermediate of the TCA cycle (Figure 1B). Thus, increased glutamine metabolism acts to replenish the TCA cycle through anaplerotic reactions, but also provides proline for collagen synthesis.

Glutamine has been shown to increase mRNA transcripts for pro-collagen I (Bellon et al., 1995), but this is not a universal finding (Ge et al., 2018). In the latter case, glutamine-derived αKG increases in pro-collagen I protein levels were contingent on activation of mTOR and consequent phosphorylation of p70-S6K and 4E-BP1 rather than changes in gene transcription (Ge et al., 2018). The formation of αKG from glutamate is reversible, meaning that αKG can also serve as a precursor in the conversion of glutamate to proline. Additionally, αKG also stabilizes intracellular collagen by promoting proline hydroxylation *via* activation of the enzyme prolyl hydroxylase (Ge et al., 2018). The relationship between αKG and proline hydroxylation is particularly interesting. Hydroxylation of prolines in hypoxia inducible factor-1 (HIF-1α) is necessary for ubiquitin-mediated proteasome degradation of this enigmatic protein. Conversely, the same post-translational modification protects intracellular collagen from being degraded in lung fibroblasts (Li and Wu, 2018), suggesting that proline hydroxylation in collagens does not create a docking site for ubiquitin binding. Finally, by acting as a co-factor for a Jumonji family histone demethylase (JMJD3), αKG promotes histone methylation at the cell survival genes X-linked IAP (XIAP) and survivin (Bai et al., 2019), thereby providing a mechanistic link between metabolism, epigenetic regulation of cell survival and resistance of fibroblasts to apoptosis.

The relative importance of changes in glycolysis and glutaminolysis is unclear. Recent data highlights that amino acids, rather than glucose, might be responsible for most of the cell mass in proliferation (Du et al., 2018), Replenishment of the TCA cycle with αKG due to a TGF-β1-induced shift toward glycolysis is logical and such a change in flux has been demonstrated in lung fibroblasts using carbon tracing (Bernard et al., 2018). Functional measures suggest that the main function of glutamine metabolism in fibroblasts is to feed biosynthetic pathways rather than ATP generation (Hamanaka et al., 2019).

## Metabolic Regulation of Fibroblast Differentiation

An interesting question has been whether metabolic reprogramming is a signal for fibroblast differentiation as well as increased activity *per se*. Not surprisingly these roles are difficult to isolate. Nevertheless, triggering of development signals like the hedgehog-Yap axis in both glycolysis (Chen et al., 2012) and glutaminolysis (Du et al., 2018) have suggested a more direct role in differentiation. Indeed, subsequent studies showed that both glutamine-depleted media and Gls inhibition prevent the differentiation of quiescent hepatic stellate cells into myofibroblasts, an effect that was not seen with glucose deprivation (Du et al., 2018).

## Fibroblast Priming

Although we have long known that fibroblasts from human fibrotic kidneys are inherently more proliferative and synthesize more collagen than their counterparts from normal kidneys (Rodemann and Muller, 1990), only recently have we started to understand this at the molecular level.

In normal cutaneous granulation tissue, completion of physiological healing is accompanied by a loss of myofibroblasts due to apoptosis (Darby et al., 1990). In pathological conditions, such as experimental pulmonary fibrosis, the persistence of fibroblasts has been shown to be due to an inherent resistance of fibroblasts to apoptosis (Huang et al., 2013). Likewise dermal fibroblasts from skin lesions in systemic scleroderma show concordant and stable gene expression differences when compared to fibroblasts isolated from healthy donors (Shin et al., 2019). In these cells, a signature of aberrant TGF-β1 signaling was sustained in isolated dermal fibroblasts maintained in culture (Shin et al., 2019). Similarly, fibrotic renal fibroblasts have upregulated responses to cytokine stimulation compared to their counterparts derived from uninjured tissue, in part due to increased cell-surface TGF-β receptor expression (Smith et al., 2017a,b).

This predisposition also extends to metabolic adaptations. Under basal conditions, skin fibroblasts derived from keloids have a higher rate of ATP synthesis than their normal skin counterparts (Vincent et al., 2008); glycolysis is the primary energy source in these cells, while normal skin fibroblasts derive their ATP mainly from oxidative phosphorylation (Vincent et al., 2008). Fibroblasts from lungs of patients with idiopathic pulmonary fibrosis (IPF) have augmented glycolysis relative to cells from normal lungs (Xie et al., 2015). In renal studies, the effect of TGF-β1 on PDC regulation was observed in fibroblasts grown from kidneys after unilateral ureteric obstruction, and not in cells from unobstructed kidneys (Smith and Hewitson, 2020), while Tian et al. have recently described a metabolic memory over multiple cell passages in left ventricular fibroblasts derived from patients with pulmonary arterial hypertension, implying an epigenetic basis (Tian et al., 2020). Finally, fibroblasts derived from patients with IPF express more Gls mRNA than their normal lung counterparts (Choudhury et al., 2020). There is however a clear heterogeneity, with some fibroblasts derived from patients with IPF (Bai et al., 2019) and systemic scleroderma (Henderson et al., 2020) more sensitive (primed) than others.

## Cellular Metabolism as a Therapeutic Target

Metabolic pathways that support persistent activation and ECM synthesis in fibroblasts may offer the opportunity for therapeutic interventions to block fibrogenesis. Such novel therapeutic strategies for fibrosis have been proposed based on proof-of-principle studies in a variety of experimental models and organs that have targeted different steps in central carbon and glutamine metabolism; 6-phosphofructo-2-kinase/fructose-2, 6-biphosphatase 3 (PFKFB3) (Xie et al., 2015) and PDC (Wei et al., 2019; Tian et al., 2020), serine-glycine synthesis (Hamanaka et al., 2018) and Gls (Cui et al., 2019), amongst others. However, although there is much *in vitro* and pre-clinical evidence, human data remains limited and indirect. Unsurprisingly, we again take our lead from cancer, where multiple small molecule inhibitors targeting cellular metabolism are under investigation in phase1/2 clinical trials [reviewed in (Akins et al., 2018)].

## Conclusions

In cancer cells, metabolic adaptations appear as prerequisites for the acquisition and maintenance of a malignant phenotype. Rapidly accumulating evidence now suggests that renal fibroblasts may likewise be metabolically reprogrammed, with glucose and glutamine consumption linked to several possible mechanisms in (myo)fibroblast activation and fibrogenesis (Figure 1). Despite the similarities with malignant cells, differences do exist, highlighting the need to define this process at an organ and cell-specific level, and to confirm both changes in metabolic flux and synthetic fate over time. Likewise, while non–renal (myo)fibroblasts offer an exciting glimpse into the metabolic adaptions in fibrosis, we eagerly await further confirmation of such adaptations in human kidney disease.

## Author Contributions

TH and ES jointly wrote and edited the manuscript. Both authors approved the manuscript for submission and agree to be accountable for its contents.

## Conflict of Interest

The authors declare that the research was conducted in the absence of any commercial or financial relationships that could be construed as a potential conflict of interest.

## Abbreviations

Gls: glutaminase
OxPhos: oxidative phosphorylation
IPF: Idiopathic pulmonary fibrosis
αSMA: α-smooth muscle actin
TGF-β1: transforming growth factor-β1.

## References

[B1] Akins N. S. Nielson T. C. Le H. V. (2018). Inhibition of glycolysis and glutaminolysis: an emerging drug discovery approach to combat cancer. Curr. Top Med. Chem. 18, 494–504. 10.2174/1568026618666180523111351 29788892 PMC6110043

[B2] Altman B. J. Stine Z. E. Dang C. V. (2016). From Krebs to clinic: glutamine metabolism to cancer therapy. Nat. Rev. Cancer 16, 619–634. 10.1038/nrc.2016.71 27492215 PMC5484415

[B3] Bai L. Bernard K. Tang X. Hu M. Horowitz J. C. Thannickal V. J.. (2019). Glutaminolysis epigenetically regulates antiapoptotic gene expression in idiopathic pulmonary fibrosis fibroblasts. Am. J. Respir. Cell Mol. Biol. 60, 49–57. 10.1165/rcmb.2018-0180OC 30130138 PMC6348715

[B4] Barcena-Varela M. Paish H. Alvarez L. Uriarte I. Latasa M. U. Santamaria E.. (2020). Epigenetic mechanisms and metabolic reprogramming in fibrogenesis: dual targeting of G9a and DNMT1 for the inhibition of liver fibrosis. Gut 70, 388–400. 10.1136/gutjnl-2019-320205 32327527

[B5] Bates J. Vijayakumar A. Ghoshal S. Marchand B. Yi S. Kornyeyev D.. (2020). Acetyl-CoA carboxylase inhibition disrupts metabolic reprogramming during hepatic stellate cell activation. J. Hepatol. 73, 896–905. 10.1016/j.jhep.2020.04.037 32376414

[B6] Bellon G. Chaqour B. Wegrowski Y. Monboisse J. C. Borel J. P. (1995). Glutamine increases collagen gene transcription in cultured human fibroblasts. Biochim. Biophys. Acta 1268, 311–323. 10.1016/0167-4889(95)00093-8 7548230

[B7] Bernard K. Logsdon N. J. Benavides G. A. Sanders Y. Zhang J. Darley-Usmar V. M.. (2018). Glutaminolysis is required for transforming growth factor-β1-induced myofibroblast differentiation and activation. J. Biol. Chem. 293, 1218–1228. 10.1074/jbc.RA117.000444 29222329 PMC5787800

[B8] Bernard K. Logsdon N. J. Ravi S. Xie N. Persons B. P. Rangarajan S.. (2015). Metabolic reprogramming is required for myofibroblast contractility and differentiation. J. Biol. Chem. 290, 25427–25438. 10.1074/jbc.M115.646984 26318453 PMC4646190

[B9] Buhl E. M. Djudjaj S. Klinkhammer B. M. Ermert K. Puelles V. G. Lindenmeyer M. T.. (2020). Dysregulated mesenchymal PDGFR-β drives kidney fibrosis. EMBO Mol. Med. 12:e11021. 10.15252/emmm.201911021 31943786 PMC7059015

[B10] Calvier L. Chouvarine P. Legchenko E. Hoffmann N. Geldner J. Borchert P.. (2017). PPARγ links BMP2 and TGFβ1 pathways in vascular smooth muscle cells, regulating cell proliferation and glucose metabolism. Cell Metab. 25, 1118–1134.e1117. 10.1016/j.cmet.2017.03.011 28467929

[B11] Chen Y. Choi S. S. Michelotti G. A. Chan I. S. Swiderska-Syn M. Karaca G. F.. (2012). Hedgehog controls hepatic stellate cell fate by regulating metabolism. Gastroenterology 143, 1319–1329.e1311. 10.1053/j.gastro.2012.07.115 22885334 PMC3480563

[B12] Chen Z. Liu M. Li L. Chen L. (2018). Involvement of the Warburg effect in non-tumor diseases processes. J. Cell Physiol. 233, 2839–2849. 10.1002/jcp.25998 28488732

[B13] Chou Y. H. Pan S. Y. Shao Y. H. Shih H. M. Wei S. Y. Lai C. F.. (2020). Methylation in pericytes after acute injury promotes chronic kidney disease. J. Clin. Invest. 130, 4845–4857. 10.1172/JCI135773 32749240 PMC7456210

[B14] Choudhary C. Kumar C. Gnad F. Nielsen M. L. Rehman M. Walther T. C.. (2009). Lysine acetylation targets protein complexes and co-regulates major cellular functions. Science 325, 834–840. 10.1126/science.1175371 19608861

[B15] Choudhary C. Weinert B. T. Nishida Y. Verdin E. Mann M. (2014). The growing landscape of lysine acetylation links metabolism and cell signalling. Nat. Rev. Mol. Cell Biol. 15, 536–550. 10.1038/nrm3841 25053359

[B16] Choudhury M. Yin X. Schaefbauer K. J. Kang J. H. Roy B. Kottom T. J.. (2020). SIRT7-mediated modulation of glutaminase 1 regulates TGF-β-induced pulmonary fibrosis. FASEB J. 34, 8920–8940. 10.1096/fj.202000564R 32519817

[B17] Cui H. Xie N. Jiang D. Banerjee S. Ge J. Sanders Y. Y.. (2019). Inhibition of Glutaminase 1 attenuates experimental pulmonary fibrosis. Am. J. Respir. Cell Mol. Biol. 61, 492–500. 10.1165/rcmb.2019-0051OC 30943369 PMC6775943

[B18] Dabiri G. Tumbarello D. A. Turner C. E. Van de Water L. (2008). Hic-5 promotes the hypertrophic scar myofibroblast phenotype by regulating the TGF-beta1 autocrine loop. J. Invest. Dermatol. 128, 2518–2525. 10.1038/jid.2008.90 18401422 PMC2597160

[B19] Darby I. Skalli O. Gabbiani G. (1990). Alpha-smooth muscle actin is transiently expressed by myofibroblasts during experimental wound healing. Lab. Invest. 63, 21–29. 2197503

[B20] Darby I. A. Hewitson T. D. (2007). Fibroblast differentiation in wound healing and fibrosis. Int. Rev. Cytol. 257, 143–179. 10.1016/S0074-7696(07)57004-X 17280897

[B21] Ding H. Jiang L. Xu J. Bai F. Zhou Y. Yuan Q.. (2017). Inhibiting aerobic glycolysis suppresses renal interstitial fibroblast activation and renal fibrosis. Am. J. Physiol. Renal. Physiol. 313, F561–f575. 10.1152/ajprenal.00036.2017 28228400

[B22] Du K. Hyun J. Premont R. T. Choi S. S. Michelotti G. A. Swiderska-Syn M.. (2018). Hedgehog-YAP signaling pathway regulates glutaminolysis to control activation of hepatic stellate cells. Gastroenterology 154, 1465–1479.e1413. 10.1053/j.gastro.2017.12.022 29305935 PMC5880682

[B23] Duffield J. S. Lupher M. Thannickal V. J. Wynn T. A. (2013). Host responses in tissue repair and fibrosis. Annu. Rev. Pathol. 8, 241–276. 10.1146/annurev-pathol-020712-163930 23092186 PMC3789589

[B24] Fukasawa H. Yamamoto T. Togawa A. Ohashi N. Fujigaki Y. Oda T.. (2004). Down-regulation of Smad7 expression by ubiquitin-dependent degradation contributes to renal fibrosis in obstructive nephropathy in mice. Proc. Natl. Acad. Sci. U.S.A. 101, 8687–8692. 10.1073/pnas.0400035101 15173588 PMC423256

[B25] Ge J. Cui H. Xie N. Banerjee S. Guo S. Dubey S.. (2018). Glutaminolysis promotes collagen translation and stability via α-Ketoglutarate-mediated mTOR activation and proline hydroxylation. Am. J. Respir. Cell Mol. Biol. 58, 378–390. 10.1165/rcmb.2017-0238OC 29019707 PMC5854958

[B26] Ghosh-Choudhary S. Liu J. Finkel T. (2020). Metabolic regulation of cell fate and function. Trends Cell Biol. 30, 201–212. 10.1016/j.tcb.2019.12.005 31983571 PMC7043867

[B27] Hamanaka R. B. Nigdelioglu R. Meliton A. Y. Tian Y. Witt L. J. O'Leary E.. (2018). Inhibition of phosphoglycerate dehydrogenase attenuates bleomycin-induced pulmonary fibrosis. Am. J. Respir. Cell Mol. Biol. 58, 585–593. 10.1165/rcmb.2017-0186OC 29019702 PMC5946329

[B28] Hamanaka R. B. O'Leary E. M. Witt L. J. Tian Y. Gokalp G. A. Meliton A. Y.. (2019). Glutamine metabolism is required for collagen protein synthesis in lung fibroblasts. Am. J. Respir. Cell Mol. Biol. 61, 597–606. 10.1165/rcmb.2019-0008OC 30973753 PMC6827066

[B29] Henderson J. Duffy L. Stratton R. Ford D. O'Reilly S. (2020). Metabolic reprogramming of glycolysis and glutamine metabolism are key events in myofibroblast transition in systemic sclerosis pathogenesis. J. Cell Mol. Med. 24, 14026–14038. 10.1111/jcmm.16013 33140521 PMC7754020

[B30] Hewitson T. D. (2009). Renal tubulointerstitial fibrosis: common but never simple. Am. J. Physiol. Renal. Physiol. 296, F1239–F1244. 10.1152/ajprenal.90521.2008 19144691

[B31] Hewitson T. D. Holt S. G. Smith E. R. (2017a). Progression of tubulointerstitial fibrosis and the chronic kidney disease phenotype—role of risk factors and epigenetics. Front. Pharmacol. 8:520. 10.3389/fphar.2017.00520 28848437 PMC5550676

[B32] Hewitson T. D. Holt S. G. Tan S. J. Wigg B. Samuel C. S. Smith E. R. (2017b). Epigenetic modifications to H3K9 in renal tubulointerstitial cells after unilateral ureteric obstruction and TGF-beta1 stimulation. Front. Pharmacol. 8:307. 10.3389/fphar.2017.00307 28611663 PMC5447091

[B33] Huang S. K. Scruggs A. M. Donaghy J. Horowitz J. C. Zaslona Z. Przybranowski S.. (2013). Histone modifications are responsible for decreased Fas expression and apoptosis resistance in fibrotic lung fibroblasts. Cell Death Dis. 4, e621–e621. 10.1038/cddis.2013.146 23640463 PMC3674355

[B34] Kaissling B. Lehir M. Kriz W. (2013). Renal epithelial injury and fibrosis. Biochim. Biophys. Acta 1832, 931–939. 10.1016/j.bbadis.2013.02.010 23466594

[B35] Kamphorst J. J. Chung M. K. Fan J. Rabinowitz J. D. (2014). Quantitative analysis of acetyl-CoA production in hypoxic cancer cells reveals substantial contribution from acetate. Cancer Metab. 2:23. 10.1186/2049-3002-2-23 25671109 PMC4322440

[B36] Kori Y. Sidoli S. Yuan Z. F. Lund P. J. Zhao X. Garcia B. A. (2017). Proteome-wide acetylation dynamics in human cells. Sci. Rep. 7:10296. 10.1038/s41598-017-09918-3 28860605 PMC5579049

[B37] Kottmann R. M. Kulkarni A. A. Smolnycki K. A. Lyda E. Dahanayake T. Salibi R.. (2012). Lactic acid is elevated in idiopathic pulmonary fibrosis and induces myofibroblast differentiation via pH-dependent activation of transforming growth factor-β. Am. J. Respir. Crit. Care Med. 186, 740–751. 10.1164/rccm.201201-0084OC 22923663 PMC3480515

[B38] Kottmann R. M. Trawick E. Judge J. L. Wahl L. A. Epa A. P. Owens K. M.. (2015). Pharmacologic inhibition of lactate production prevents myofibroblast differentiation. Am. J. Physiol. Lung Cell Mol. Physiol. 309, L1305–1312. 10.1152/ajplung.00058.2015 26408551 PMC4669339

[B39] Kwan B. Fuhrer T. Zhang J. Darshi M. Van Espen B. Montemayor D.. (2020). Metabolomic markers of kidney function decline in patients with diabetes: evidence from the Chronic Renal Insufficiency Cohort (CRIC) study. Am. J. Kidney Dis. 76, 511–520. 10.1053/j.ajkd.2020.01.019 32387023 PMC7529642

[B40] Leaf I. A. Duffield J. S. (2017). What can target kidney fibrosis? Nephrol. Dial. Transplant. 32, i89–i97. 10.1093/ndt/gfw388 28391346

[B41] Li P. Wu G. (2018). Roles of dietary glycine, proline, and hydroxyproline in collagen synthesis and animal growth. Amino Acids 50, 29–38. 10.1007/s00726-017-2490-6 28929384

[B42] Li Q. Qin Z. Nie F. Bi H. Zhao R. Pan B.. (2018). Metabolic reprogramming in keloid fibroblasts: aerobic glycolysis and a novel therapeutic strategy. Biochem. Biophys. Res. Commun. 496, 641–647. 10.1016/j.bbrc.2018.01.068 29337061

[B43] Lundby A. Lage K. Weinert B. T. Bekker-Jensen D. B. Secher A. Skovgaard T.. (2012). Proteomic analysis of lysine acetylation sites in rat tissues reveals organ specificity and subcellular patterns. Cell Rep. 2, 419–431. 10.1016/j.celrep.2012.07.006 22902405 PMC4103158

[B44] Meng X. M. Nikolic-Paterson D. J. Lan H. Y. (2016). TGF-beta: the master regulator of fibrosis. Nat. Rev. Nephrol. 12, 325–338. 10.1038/nrneph.2016.48 27108839

[B45] Negmadjanov U. Godic Z. Rizvi F. Emelyanova L. Ross G. Richards J.. (2015). TGF-beta1-mediated differentiation of fibroblasts is associated with increased mitochondrial content and cellular respiration. PLoS ONE 10:e0123046. 10.1371/journal.pone.0123046 25849590 PMC4388650

[B46] Nigdelioglu R. Hamanaka R. B. Meliton A. Y. O'Leary E. Witt L. J. Cho T.. (2016). Transforming growth factor (TGF)-beta promotes *de novo* serine synthesis for collagen production. J. Biol. Chem. 291, 27239–27251. 10.1074/jbc.M116.756247 27836973 PMC5207151

[B47] O'Neill L. A. Kishton R. J. Rathmell J. (2016). A guide to immunometabolism for immunologists. Nat. Rev. Immunol. 16, 553–565. 10.1038/nri.2016.70 27396447 PMC5001910

[B48] Para R. Romero F. George G. Summer R. (2019). Metabolic reprogramming as a driver of fibroblast activation in pulmonary fibrosis. Am. J. Med. Sci. 357, 394–398. 10.1016/j.amjms.2019.02.003 30879738 PMC6478549

[B49] Pena M. J. Lambers Heerspink H. J. Hellemons M. E. Friedrich T. Dallmann G. Lajer M.. (2014). Urine and plasma metabolites predict the development of diabetic nephropathy in individuals with Type 2 diabetes mellitus. Diabet. Med. 31, 1138–1147. 10.1111/dme.12447 24661264

[B50] Rodemann H. P. Muller G. A. (1990). Abnormal growth and clonal proliferation of fibroblasts derived from kidneys with interstitial fibrosis. Proc. Soc. Exp. Biol. Med. 195, 57–63. 10.3181/00379727-195-43118 2399261

[B51] Shin J. Y. Beckett J. D. Bagirzadeh R. Creamer T. J. Shah A. A. McMahan Z.. (2019). Epigenetic activation and memory at a TGFB2 enhancer in systemic sclerosis. Sci. Transl. Med. 11:eaaw0790. 10.1126/scitranslmed.aaw0790 31217334 PMC6995475

[B52] Si M. Wang Q. Li Y. Lin H. Luo D. Zhao W.. (2019). Inhibition of hyperglycolysis in mesothelial cells prevents peritoneal fibrosis. Sci. Transl. Med. 11:aav5341. 10.1126/scitranslmed.aav5341 31167927

[B53] Sivanand S. Viney I. Wellen K. E. (2018). Spatiotemporal control of Acetyl-CoA metabolism in chromatin regulation. Trends Biochem. Sci. 43, 61–74. 10.1016/j.tibs.2017.11.004 29174173 PMC5741483

[B54] Smith E. R. Hewitson T. D. (2020). TGF-beta1 is a regulator of the pyruvate dehydrogenase complex in fibroblasts. Sci. Rep. 10:17914. 10.1038/s41598-020-74919-8 33087819 PMC7578649

[B55] Smith E. R. Holt S. G. Hewitson T. D. (2017a). FGF23 activates injury-primed renal fibroblasts via FGFR4-dependent signalling and enhancement of TGF-beta autoinduction. Int. J. Biochem. Cell Biol. 92, 63–78. 10.1016/j.biocel.2017.09.009 28919046

[B56] Smith E. R. Tan S. J. Holt S. G. Hewitson T. D. (2017b). FGF23 is synthesised locally by renal tubules and activates injury-primed fibroblasts. Sci. Rep. 7:3345. 10.1038/s41598-017-02709-w 28611350 PMC5469734

[B57] Smith E. R. Wigg B. Holt S. Hewitson T. D. (2019). TGF-beta1 modifies histone acetylation and acetyl-coenzyme A metabolism in renal myofibroblasts. Am. J. Physiol. Renal. Physiol. 316, F517–F529. 10.1152/ajprenal.00513.2018 30623724

[B58] Spange S. Wagner T. Heinzel T. Kramer O. H. (2009). Acetylation of non-histone proteins modulates cellular signalling at multiple levels. Int. J. Biochem. Cell. Biol. 41, 185–198. 10.1016/j.biocel.2008.08.027 18804549

[B59] Sutendra G. Kinnaird A. Dromparis P. Paulin R. Stenson T. H. Haromy A.. (2014). A nuclear pyruvate dehydrogenase complex is important for the generation of acetyl-CoA and histone acetylation. Cell 158, 84–97. 10.1016/j.cell.2014.04.046 24995980

[B60] Tian L. Wu D. Dasgupta A. Chen K. H. Mewburn J. Potus F.. (2020). Epigenetic metabolic reprogramming of right ventricular fibroblasts in pulmonary arterial hypertension: a pyruvate dehydrogenase kinase-dependent shift in mitochondrial metabolism promotes right ventricular fibrosis. Circ. Res. 126, 1723–1745. 10.1161/CIRCRESAHA.120.316443 32216531 PMC7274861

[B61] Tomasek J. J. Gabbiani G. Hinz B. Chaponnier C. Brown R. A. (2002). Myofibroblasts and mechano-regulation of connective tissue remodelling. Nat. Rev. Mol. Cell. Biol. 3, 349–363. 10.1038/nrm809 11988769

[B62] Venkatachalam M. A. Weinberg J. M. (2015). Fibrosis without fibroblast TGF-beta receptors? Kidney Int. 88, 434–437. 10.1038/ki.2015.170 26323068 PMC5773454

[B63] Vincent A. S. Phan T. T. Mukhopadhyay A. Lim H. Y. Halliwell B. Wong K. P. (2008). Human skin keloid fibroblasts display bioenergetics of cancer cells. J. Invest. Dermatol. 128, 702–709. 10.1038/sj.jid.5701107 17943178

[B64] Wei Q. Su J. Dong G. Zhang M. Huo Y. Dong Z. (2019). Glycolysis inhibitors suppress renal interstitial fibrosis via divergent effects on fibroblasts and tubular cells. Am. J. Physiol. Renal. Physiol. 316, F1162–f1172. 10.1152/ajprenal.00422.2018 30969803 PMC6620587

[B65] Weinert B. T. Narita T. Satpathy S. Srinivasan B. Hansen B. K. Schölz C.. (2018). Time-resolved analysis reveals rapid dynamics and broad scope of the CBP/p300 acetylome. Cell 174, 231–f 244.e212. 10.1016/j.cell.2018.04.033 29804834 PMC6078418

[B66] Wu H. Kirita Y. Donnelly E. L. Humphreys B. D. (2019). Advantages of single-nucleus over single-cell RNA sequencing of adult kidney: rare cell types and novel cell states revealed in fibrosis. J. Am. Soc. Nephrol. 30, 23–32. 10.1681/ASN.2018090912 30510133 PMC6317600

[B67] Xie N. Tan Z. Banerjee S. Cui H. Ge J. Liu R. M.. (2015). Glycolytic reprogramming in myofibroblast differentiation and lung fibrosis. Am. J. Respir. Crit. Care Med. 192, 1462–1474. 10.1164/rccm.201504-0780OC 26284610 PMC4731722

[B68] Yin X. Choudhury M. Kang J. H. Schaefbauer K. J. Jung M. Y. Andrianifahanana M.. (2019). Hexokinase 2 couples glycolysis with the profibrotic actions of TGF-β. Sci. Signal. 12:eaax4067. 10.1126/scisignal.aax4067 31848318

[B69] Zhao X. Kwan J. Y. Y. Yip K. Liu P. P. Liu F. F. (2020). Targeting metabolic dysregulation for fibrosis therapy. Nat. Rev. Drug Discov. 19, 57–75. 10.1038/s41573-019-0040-5 31548636

